# Pathogenesis of Epilepsy: Challenges in Animal Models

**Published:** 2013-11

**Authors:** Yow Hui Yin, Nurulumi Ahmad, Mohd Makmor-Bakry

**Affiliations:** 1 Faculty of Pharmacy, University Kebangsaan Malaysia, Kuala Lumpur, Malaysia

**Keywords:** Animal models, Epileptogenesis, Pathogenesis of epilepsy, Temporal lobe epilepsy

## Abstract

Epilepsy is one of the most common chronic disorders affecting individuals of all ages. A greater understanding of pathogenesis in epilepsy will likely provide the basis fundamental for development of new antiepileptic therapies that aim to prevent the epileptogenesis process or modify the progression of epilepsy in addition to treatment of epilepsy symptomatically. Therefore, several investigations have embarked on advancing knowledge of the mechanism underlying epileptogenesis, understanding in mechanism of pharmacoresistance and discovering antiepileptogenic or disease-modifying therapy. Animal models play a crucial and significant role in providing additional insight into mechanism of epileptogenesis. With the help of these models, epileptogenesis process has been demonstrated to be involved in various molecular and biological pathways or processes. Hence, this article will discuss the known and postulated mechanisms of epileptogenesis and challenges in using the animal models.

## Introduction

Epilepsy is a neurological disorder characterized by recurrent and unpredictable interruptions of normal brain function, called epileptic seizure (1). It is one of the most common chronic disorders affecting around 50 million individuals of all ages worldwide (2). The median prevalence of life-time epilepsy for developed countries was 5.8 per 1,000 and for developing countries up to 15.4 per 1,000 (3). The median prevalence of active epilepsy despite treatment was 4.9 per 1,000 for developed countries and up to 12.7 per 1,000 for developing countries (3). This showed that prevalence of epilepsy is higher in developing countries compared to developed countries. In Asia, the median life-time prevalence is estimated at 6 per 1,000, which is lower than in developing countries in other areas of the world (4).

According to the causal etiology, epilepsy can be divided into 3 categories: idiopathic, acquired (symp-tomatic) and cryptogenic (presumed symptomatic) (5-7). Idiopathic epilepsy is epilepsy without underlying structural brain lesion or other neurologic signs or symptoms, which is presumed to be genetic and generally has onset during childhood. Acquired epilepsy is epileptic seizures as a result of one or more identifiable structural lesions of the brain. Cryptogenic epilepsy refers to the epilepsy that is believed to be symptomatic, with unidentifiedcause (5, 6). Among the epilepsy cases, approximately 40% have known etiology (8), including traumatic brain injury, ischemic stroke, intracerebral hemorrhage, central nervous system infections, brain tumors, several neurodegenerative diseases, and prolonged acute symptomatic seizures such as complex febrile seizures or status epilepticus (SE) (6,7,9).

On the other hand, the International League against Epilepsy has classified seizures into two major types namely generalized seizures which involve both hemispheres of brain and partial (focal) seizures which begin locally in one hemisphere of the brain (10). Majority of patients with epilepsy suffer from partial seizures (11). Temporal lobe epilepsy (TLE) is the most common and difficult-to-treat type of partial epilepsy (10, 11). This may be attributed to temporal lobe structures, typically the hippocampus, the amygdala and the piriform cortex which are most susceptible to epileptogenesis-triggering brain insult (12). Therefore, TLE is commonly investigated in order to understand the mechanism underlying epileptogenesis, antiepileptic pharmacoresistance and to discover antiepileptogenic or disease-modifying therapy. Animal models of epilepsy have been suggested to play important roles in these mechanistic studies (13, 14). Hence, this review article discusses the known and postulated mechanisms of epileptogenesis and challenges in using the animal models in epilepsy study. The databases that were used for literature searching included Science direct, PubMed and Wiley Online Library. The search keywords included pathogenesis of epilepsy, epileptogenesis, animal models, neurotransmission pathway, channelopathies, neurogenesis and rewiring pathway, inflammatory pathway, apoptotic pathway, gene regulation and other related keywords. Only articles published in English were reviewed.

**Pathogenesis of epilepsy**

*Definition of epileptogenesis*

**Figure 1:**
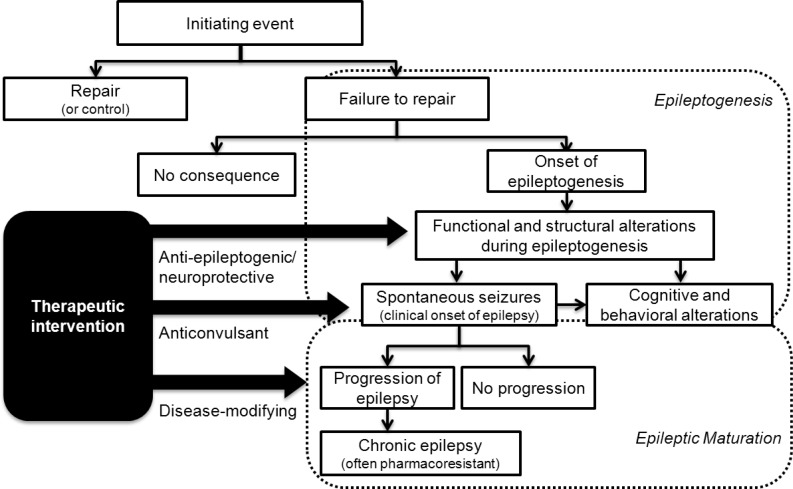
Steps in the development and progression of epilepsy and possible therapeutic interventions (Adapted from (11) with permission)

Currently, there is no universally accepted definition for epileptogenesis. The term epilepto-genesis is defined as a process that leads to the occurrence of the first spontaneous seizure and recurring epileptiform events after the brain insult (Figure 1) (15). Latency period refers to seizure-free or pre-epileptic periods between the brain insult and the occurrence of the first spontaneous seizure (15, 16). There is evidence that neurobiological changes that occur during the latency period continue to progress even after diagnosis of epilepsy and contribute to its progression (17, 18). Hence, the proposed definition for epileptogenesis includes processes that involve both development and progression of epilepsy (19). Nevertheless, recently, Sloviter Bumanglag proposed and defined secondary changes or progression process after epileptogenesis as ‘epileptic maturation’. They reviewed ‘epileptogenesis’ and ‘epileptic maturation’ as two distinct processes (15).

*Mechanism of epileptogenesis*

Epileptogenesis can involve various biological pathways or processes, structural and functional changes. In general, it is unclear which mechanisms are required or necessary for the genesis of epilepsy. However, several experimental studies have provided some insights into the actual and postulated mechanisms of epileptogenesis. 

**Neurotransmission signaling pathway**

Glutamate and γ-aminobutyric acid (GABA) are the two neurotransmitters that have been studied extensively in relation to epilepsy. Both glutam-atergic and GABAergic system play crucial roles in epileptic phenomena. It has been hypothesized that the neuronal hyperexcitability in epilepsy is due to imbalance between glutamate-mediated excitation and GABA-mediated inhibition (12). Glutamate is a main excitatory neurotrans-mitter in brain that is responsible for generating excitatory postsynaptic potentials by depolarizing the neurons (20, 21). Generally, glutamate receptors are classified into ionotropic (ligand-gated cation channels) receptors: α-amino- 3- hydroxy-5-methyl-isoxazole-4-propionic acid (AMPA), N-methyl-D-aspartic acid (NMDA) and kainate, and metabotropic (G protein-coupled) receptors (20-22). Glutamatergic molecular mecha-nisms that are involved during initiation and progression of epilepsy include upregulation of glutamate receptors (23-25), elevation in extra-cellular glutamate concentration (26, 27), abnormalities in glutamatergic transporters (27, 28) and autoimmune mechanism (29). These mechan-isms contribute to excessive glutamatergic activity, which plays an important role in hyperexcitability and epilepsy (12). This phenomenon recorded as an interictal spike on electroencephalogram, known as ‘paroxysmal depolarizing shift’ is intracellularly associated with epileptic discharges in neurons (20, 30, 31). Paroxysmal depolarizing shift is associated with depolarization due to a giant excitatory synaptic potential with characteristic of burst discharge, which is dependent on activation of AMPA receptors as initial components and NMDA receptors as later components (30).

Conversely, GABA is recognized as the main inhibitory neurotransmitter, which generates inhibitory presynaptic potentials by hyperpolarizing the neurons (32, 33). GABAergic system has an important role in counter-balancing the neuronal excitation and therefore suppressing the epileptiform discharges (32). There are two types of GABA receptors that are involved in pathogenesis of epilepsy, namely GABA_A_ and GABA_B_ receptors. GABA_A_ receptors (ligand-gated ion channels) mediate rapid inhibitory presynaptic potentials by increasing influx of chloride, and GABA_B_ receptors (G-protein-coupled receptors) mediate slow inhibitory presynaptic potentials by increasing the potassium conductance and decreasing the calcium entry (33-35). It is hypothesized that reduction or loss of GABAergic inhibition may increase the probability of generating excitatory postsynaptic potentials and synchronizing burst discharges, and therefore induce epileptogenesis (12, 31). The GABAergic mechanisms that have been proposed include impairment of GABA release (36), changes in GABA receptors (37, 38), impairment of GABA synthesis (39, 40) and neuronal loss (41, 42). 

Other neurotransmitters such as serotonin, noradrenaline and dopamine also play a role in epileptic mechanism. Serotonin, also known as 5-hydroxytryptamine is a monoamine neurotrans-mitter that is derived from amino acid tryptophan. There are several serotonin receptor subtypes expressed in the central nervous system, such as 5-HT_1A_, 5-HT_2C_ and 5-HT_7_. Receptors are present on cortical and/ or hippocampal neurons (43, 44). Experimental data from animal models and humans reveal that serotonergic neurotransmission is significantly involved in pathogenesis of epilepsy: depletion of brain serotonin in genetically epilepsy-prone rat model of audiogenic seizures (45, 46); lower seizure thresholds in mutants mice lacking 5-HT_1A_ or 5-HT_2C_ receptor subtype (47, 48); decrease in 5-HT_1A_ receptor binding in epileptogenic zone of TLE patients (49). The serotonergic system in modulating neuronal excitability has been complicated by diversity of serotonin receptor subtypes. Generally, neuronal excitability can be reduced during hyperpolarization of glutamatergic neurons by 5-HT_1A_ receptors, depolarization of GABAergic neurons by 5-HT_2C_ receptors and inhibition of 5-HT_3_ and 5-HT_7_ receptors (44). 

Noradrenaline is a catecholamine produced from dopamine, which is released either as a hormone from adrenal medulla or as a neurotransmitter in the central and sympathetic nervous systems from noradrenergic neurons (50). Multiple studies demonstrate that endogenous noradrenaline has an anticonvulsant role in epilepsy. These include noradrenaline depletion increased susceptibility to seizure induction (51, 52) and noradrenaline loss increased neuronal damage in various limbic regions of rats after seizure induction (53). It is postulated that the protective effect of noradrenaline is attributed to counteraction of an epileptic circuit formation and modification in the epilepsy-induced neuronal changes (54).

Another catecholamine neurotransmitter, dopam-ine, exerts an ambiguous and complex pathway in pathogenesis of epilepsy. Researchers have found that dopaminergic pathway is associated with pathophysiology of two idiopathic epilepsies, i.e. autosomal dominant nocturnal frontal lobe epilepsy with significant reduction in dopamine D1 receptor binding (55) and juvenile myoclonic epilepsy with decrease in binding potential to the dopamine transporter (56). This is consistent with the hypothesis that decrease in inhibitory dopaminergic activity predisposes to hyperexcitability and epilepsy (57). However, activation of different dopamine receptor families (D1 and D2) may produce diverging effects on neuronal excitability, in which D1 receptor has proconvulsant effect (58) and D2 receptor has anticonvulsant effect (58-60). A recent study in TLE patients found that there was a decrease in dopamine D2/D3 receptor binding in epileptogenic zone of these patients (61). These evidences show that dopamine might play a specific role in modulating seizures. 

**Molecular and genetic mechanisms: Ion channels and receptors**

Recent advances in genetics and molecular biology have demonstrated that several epilepsy syndromes are attributed to mutations in genes encoding ion channel proteins that lead to hyperexcitability of neurons (31, 62, 63). Channelopathy is a term used to describe ion channel dysfunction or defect (63). Ion channels are pore-forming proteins along the lipid membrane of cells that allow movement of selected ions across cell membranes to maintain negative resting membrane potential inside the cells (63, 64). There are two types of ion channels, which are voltage-gated channels controlled by changes in membrane potential and ligand-gated channels that are activated by ligand binding such as GABA and acetylcholine neurotransmitters (63). Ion channels are involved in generating electric currents via ion charges. Generally, cation channels mainly generate action potentials and contribute to neuronal excitability, in contrast, anion channels are involved in inhibitory mechanism for neuronal excitatory process (65). Hence, it is hypothesized that in imbalance of ion charges due to channelopathies, either anion or cation channel can induce epileptogenesis (65).

Channelopathies are key factors of pathogenesis in human epilepsy, predominantly in idiopathic epilepsy (66, 67). Mutations in genes expressing channels of potassium, sodium, chloride, calcium and receptors of acetylcholine and GABA have been reported in idiopathic epilepsy (Table 1) (63, 68). In addition, channelopathies can also be the pathogenesis in acquired epilepsy due to secondary changes in ion channels via transcriptional and post-translational mechanisms (66, 67). 

On the other hand, recent studies have found that channelopathy involves the hyperpolarization-activated cyclic nucleotide gated (HCN) channels that may contribute to TLE (69) and absence seizure (70). HCN channels are voltage-gated ion channels that conduct the hyperpolarization-activated cationic current, *I*_h_, which regulate resting membrane potential of neurons (71, 72). HCN channels are activated by membrane hyperpolarization and lead to inhibitory effects of *I*_h_ on neuronal excitability (71, 72). Experimental animal model studies have shown that down regulation of HCN channels and loss of channel expression subsequently cause a reduction in *I*_h_ density, and ultimately contribute to neuronal hyperexcitability (69, 73). Hence, HCN channelo-pathy may play a role in epileptogenesis.

**Neurogenesis and rewiring pathway: Structural, neurochemical and cellular changes**

Aberrant hippocampal neurogenesis, a process of new neurongeneration, and new circuit creation has been proposed as another important pathogenesis in epilepsy (74, 75). Various changes include structural, neurochemical and cellular changes which may occur following acute seizures in patients with brain insults (76). 

Multiple structural alterations in the hippocampus could occur after acute seizures, including degeneration of dentate hilar neurons and CA1 – CA3 pyramidal neurons, aberrant sprouting and synaptogenesis of mossy fibers and loss of inhibitory GABAergic interneurons (76). Mossy fiber sprouting involves synaptic reorganization of mossy fibers, which are axons of dentate granule cells for forming new synaptic contacts with an abnormal location, the inner third molecular layer of dentate gyrus or the supragranular area (77, 78). This structural reorga-nization would be attributable to synapse elimination due to neuronal death of mossy cells, which are principal excitatory neurons in dentate hilus that normally project to the supragranular area (78, 79). As a consequence of axon sprouting and synaptogenesis in mossy fiber pathway, the new neuronal circuitry forms a recurrent excitatory circuit in dentate granule cells that is expected to increase excitatory drive and eventually promote epileptogenesis (78, 79). Another proposed hypo-thesis of structural reorganization is the functional disconnection of dormant basket (inhibitory) cells with excitatory neurons. This hypothesis implies that GABAergic interneurons (basket cells) survive after an epileptogenic injury, but they remain in dormant state and are unable to provide feedback inhibition to granule cells due to seizure-induced death of major excitatory neurons) mossy cells( that results in reducing excitatory drive on these basket cells (80, 81). Dormancy of basket cells leads to loss of inhibition, possibly contributing to epileptogenesis.

**Table 1:** Channelopathies in idiopathic epilepsy (62, 68)

Epilepsy phenotype	Channel (Gene involved)					
	Sodium	Potassium	Chloride	Calcium	GABA	Acetylcholine
Autosomal dominant nocturnal frontal lobe epilepsy						CHRNA4, CHRNB2
Benign familial neonatal infantile seizures	SCN2A					
Benign familial neonatal seizures		KCNQ2,KCNQ3				
Childhood absence epilepsy			CLCN2	CACNA1H	GABRG2	
Epilepsy with grand mal seizures on awakening			CLCN2			
Episodic ataxia type 1		KCNA1				
Episodic ataxia type 2				CACNA1A, CACNB4		
Familial hemiplegic migraine				CACNA1A		
Febrile seizures					GABRG2	
Generalized epilepsy with febrile seizures plus	SCN1A, SCN2A, SCN1B				GABRG2	
Generalized epilepsy with paroxysmal dyskinesia		KCNMA1				
Infantile spasms	SCN1A					
Intractable childhood epilepsy with generalized tonic-clonic seizures	SCN1A					
Juvenile absence epilepsy			CLCN2			
Juvenile myoclonic epilepsy			CLCN2		GABRA1,GABRD	
Myokymia		KCNQ2				
Severe myoclonic epilepsy of infancy	SCN1A				GABRG2	
Spinocerebellar ataxia type 6				CACNA1A		

Apart from structural changes, acute seizures can also up regulate several neurotropic factors and other proteins in the hippocampus. These include nerve growth factor (82, 83), brain-derived neuro-trophic factor (82, 83), fibroblast growth factor-2 (84), vascular endothelial growth factor (VEGF) (85) and sonic hedgehog (86). The neurotrophins, i.e. nerve growth factor, brain-derived neurotrophic factor and fibroblast growth factor-2, have a role in neuronal survival, differentiation, growth, synaptic plasticity and excitability (32). VEGF induces angiogenesis, but increase in VEGF could contribute to blood brain barrier disruption and inflammation in brain (85). On the other hand, sonic hedgehog is a secreted protein that regulates the proliferation and survival of neuronal and glial precursors (86). Up-regulation of these proteins might contribute to neurogenesis in hippocampus (76). 

Cellular changes are also involved in the hippo-campus following acute seizures. These changes include increase of neurogenesis, abnormal migra-tion of newly born granule cells into dentate hilus and dentate molecular layer, and occurrence of hilar basal dendrites in newly added granule cells (74-76). All these changes might form an aberrant circuitry that contributes to generation of epileptiform activity by creating excitatory loops and thus enhance seizure initiation and propagation.

**Immunological and inflammatory pathway**

Cytokines are polypeptide mediators that are associated with activation of immune system and inflammatory reactions (87). Recent studies in animal models have shown that inflammatory cytokines are involved in the pathogenesis of epilepsy. The inflammatory cytokines such as interleukin (IL)-1β, IL-6 and tumor necrosis factor-α have been shown to be up-regulated and over-expressed in brain regions involved in generating and propagating epileptic activity (88-90). Glial cells, particularly microglia and astrocytes, the non-neuronal cell components in central nervous system, are known to be sources for the inflammatory cytokines in the epileptic tissues (90-92). Hence, glial cells play a role in regulating immune or inflammatory response during epileptogenesis. The release of inflammatory cytokines in microglia and astrocytes is usually followed by a cascade of down-stream inflammatory events which can recruit neurons and activate adaptive immune system (93, 94).

However, there is concern on how the activated inflammatory pathway contributes to pathogenesis of epilepsy. It has been revealed that these inflammatory cytokines have deleterious effects on neurons via alteration of neuronal excitability, production of toxic mediators and increase impermeability of blood-brain barrier (BBB) (91,94). IL-1β can induce the activation of NMDA receptor, thus enhancing NMDA-mediated ion calcium influx into neurons and ultimately promoting neuronal hyperexcitability (95, 96). Similar to IL-1β, tumor necrosis factor-α can also induce neuronal excitability via up-regulation of AMPA receptors which favors the ion calcium influx into neuron and down-regulation of GABA receptors in which the inhibitory synapse strength decreases(97). Apart from excitotoxic effects, inflammatory cytokines may contribute to apoptotic neuronal death, which is likely due to production of neurotoxic mediators and NMDA- and AMPA-mediated glutamatergic excitotoxicity (95). Besides, inflammation reactions may alter the BBB permeability (90, 91). The BBB disruption may induce epileptogenesis by uptake of serum albumin into astrocytes via binding to transforming growth factor-β receptor and triggering subsequent events that contribute to neuronal hyperexcitability and eventually epileptiform activity (98, 99).

In addition, Febene and colleagues (100) showed that inflammatory cell adhesion has a role in seizure pathogenesis. They showed that expression of vascular cell adhesion molecules is elevated and leukocyte adhesion to endothelial cells is enhanced in cerebral blood vessels, which is mediated by leukocyte mucin P-selectin glycoprotein ligand-1 and leukocyte integrins after pilocarpine-induced seizure (100). Consequently, this results in a cascade of events including increased leukocyte extravasation, cerebral inflammation, BBB leakage, enhance neuro-nal excitatory and ultimately epileptogenesis (100).

**Apoptotic pathway**

Apoptosis is a programmed cell death process during normal growth and development in multicellular organisms for maintaining cell homeostasis (101). Experimental and clinical data have shown that significant neuronal cell loss occurs after brain insults and apoptotic pathway may be involved in this cell loss in addition to other mechanisms such as excitatory glutamate-mediated toxicity (102-105). There are two major families of genes that regulate the apoptosis pathway in mammals: caspases and Bcl-2 family proteins (106). Caspases are a family of cysteine proteases and mainly function as apoptotic initiator (caspases 2, 8, 9, 10) or executioner (caspases 3, 6, 7) in apoptosis process (102). Bcl-2 family proteins are important regulators of the apoptosis process in cellular life and death decision. This characteristic is attributed to its anti-apoptotic (e.g. Bcl-2, Bcl-XL, Bcl-W and Mcl-1) and pro-apoptotic members (e.g. Bax, Bak, Bad, Bid and other BH-3 only proteins) (102, 107). Apart from caspases and Bcl-2, other proteins such as p53, tumor necrosis factor, Fas ligand and nuclear factor-κB are also essential in regulating the cell death mechanisms (102, 108). 

In experimental models of epilepsy and epilepto-genesis, researchers have revealed that executioner caspase-3 and -6 are activated and actively expressed in the hippocampus (103, 109, 110). Be-sides, Bcl-2 family proteins such as Bax and Bcl-2 are also involved in pathogenesis of human temporal lobe epilepsy models (102, 106, 111). These eviden-ces suggested that apoptotic pathway may play a role in the pathogenesis of epilepsy. 

**Gene and protein regulation**

Several studies have demonstrated that alteration in gene expression is triggered after brain insults and have proposed that this might be regulated by transcription factors. Cellular immediate early genes or inducible transcription factors such as members of the Jun family (c-*jun*, *jun*B, *jun*D) and the Fos family (c-*fos*, *fos*B and fos-related antigenes*fra-1* and *fra-2*) are believed to be involved in pathogenesis of seizures (112). Both of these gene families encode transcription factors c-*fos* and c-*jun*, which are the major components of the transcription factor activator protein-1 (113). Early up-regulation and expression of c-*fos* and c-*jun* mRNA have been demonstrated in hippocampal neurons during experimental seizures (114, 115) or cerebral ischemia (116). The specific roles of IEGs immediate early genes in epileptogenesis have not been elucidated. Generally, in central nervous system, transcription factors of Fos and Jun families are involved in gene transcription, cell proliferation, regeneration and cell death (112, 113, 117, 118). The expression of immediate early genes may partly form the biological cascade that induces apoptotic cell death of neurons (117).

Another transcription factor, inducible cyclic adenoside monophosphate (cAMP) early repressor (ICER) also plays a role in epileptogenesis. ICER is a group of proteins produced from the cAMP-responsive element modulator (CREM) gene and ICER messenger RNAs that are transcribed by an internal promoter of the CREM (119). ICER serves as an endogenous repressor of cAMP-responsive element (CRE)-mediated gene transcription (120, 121). With this characteristic, ICER plays a role as important transcriptional regulator of neuronal plasticity and apoptosis in nervous system by repressing CRE-mediated gene transcription and antagonizing activity of cAMP-responsive element binding protein (CREB) transcription factor. CREB is an activator for CRE transcription that is crucial for neuronal survival (120, 121). Up-regulation of ICER expression in neurons after excitotoxic stimuli and over-expression of ICER in cultured neurons have shown apoptotic effect (122). Controversially, recent studies have suggested that high expression of ICER suppresses the kindling process in experimental animal models (123, 124). Therefore, the mechanism of ICER in epileptogenesis still remains unclear. 

Changes of neuropeptides expression in hippo-campus following seizure have been observed in experimental studies. These neuropeptides include neuropeptide Y (NPY), somatostatin, cholecysto-kinin, neurokinin B, galanin, thyrotropin-releasing hormone and cortistatin (125-127). In hippocampus, NPY is co-localized in GABAergic interneurons and hence, it has inhibitory actions on neuronal excitation (128, 129). Over-expression of NPY and its mRNA in hippocampus (125,126,130) and aberrant expression of NPY in hippocampal granule cells and mossy fibers that normally do not contain this peptide (130, 131) have been reported in experi-mental seizure studies. It has been suggested that these findings represent endogenous adaptive mechanism to counteract the hyperexcitability state during seizure stimulation (129). Collaterally, this concept is supported by studies that showNPY gene therapy in animal models reduced the spontaneous seizure and delayed the progression of seizure (132, 133).

Another neuropeptide, galanin also has been demonstrated to be involved in modulating seizure activity (134). Galanin is widely distributed throughout the central nervous system and is involved in various brain functions (135). It is well-known as a universal neurotransmitter inhibitor, by inhibiting the release of neurotransmitters including glutamate, acetylcholine and noradrenaline (135). In other words, it serves as a seizure modulator by restoring the balance between glutamatergic excitation and galaninergic inhibition in dentate gyrus of hippocampus (135) through the activation of galanin receptors GalR1 and GalR2 (136, 137). Depletion of stored galanin in dentate hilus has been observed after exposure to seizure stimuli, interestingly the galanin expression reappeared and increased a few hours after the stimulation (134). In addition, studies on over-expression of galanin in animal models have shown that galanin played a significant role in decreasing the susceptibility of seizure during seizure induction (138). The available evidences supported the role of gene and protein regulation in epileptogenesis.

**Animal models for epileptogenesis**

A variety of animal models have been developed to study epilepsy and epileptic seizures. Each animal model demonstrates different types of epilepsy (Table 2) (14, 44).However, each model is unique for a specific study purpose (14, 139). The objective of each experiment is essential for selection of a suitable animal model. For example, mechanism of epileptogenesis can be explored using several models such as kindling, post-SE with spontaneous recurrent seizure, traumatic brain injury-induced epilepsy, stroke-induced epilepsy and febrile seizure models (8, 140). In addition, genetic animal models of generalized epilepsy, such as tottering mice with spontaneous recurrent seizures and genetically epilepsy prone rats with reflex seizures can also be employed (8, 141). Nevertheless, not all these models are suitable for the testing antiepileptogenic or disease-modifying therapies (139). The National Institutes of Health/National Institute of Neurological Disorders and Stroke Workshop recommended only two models as useful for antiepileptogenic treatment discovery, which are kindling and post-SE models with spontaneous recurrent seizure (139). 

Kindling is a model of chronic seizures which involves progressive intensification of brain excitability by repeated excitatory stimuli (electrical or chemical) that ultimately induce seizure disorder (14, 142). Electrical kindling usually stimulates a specific brain region, such as amygdala, hippocampus or other brain regions, via chronically implanted depth electrodes (32, 142, 143). Chemical kindling, such as pentylenetetrazoleis ultilized in some studies, but this method has been much less utilized than electrical kindling (144, 145). Pentylenetetrazole kindling involves repeated injection of pentylenetetrazole to cause gradual seizure development as a result of which a significant neuronal loss in hippocampus CA1 and CA3 structures have been observed (144). Kindling model is widely used as a model of TLE because the fully kindled seizures resemble the complex partial seizures and secondarily generalized seizures (32, 143). 

**Table 2:** Experimental animal models and types of epilepsy (14, 44)

Type of epilepsy	Animal models*
*Partial seizures*	
Acute seizures	Electrical stimulation, e.g. 6-Hz
Chronic seizures	Electrical or chemical kindling Topical chemoconvulsants, which block inhibition, e.g. penicillin, bicuculline, picrotoxin, pentylentetrazol, strychnine Topical chemoconvulsants, which enhance excitation, e.g. carbachol, kainate Freeze lesion or partially isolated cortical slab Implanted metals, e.g. Al_2_O_3_, cobalt Experimental febrile seizures Post-traumatic epilepsy (PTE) induced by lateral fluid percussion brain injury Hippocampal sclerosis model, e.g. kainic acid, pilocarpine, post-status epilepticus models Focal dysplasia model, e.g. neonatal freeze, prenatal radiation, methylazoxymethanol
Post-status epilepticus models with spontaneous recurrent seizures	Electrical status epilepticus induction, e.g. perforanth path, basolateral amygdala Chemical status epilepticus induction, e.g. pilocarpine, kainate
*Generalized seizures*	
Generalized tonic-clonic seizures	Electrical stimulation, e.g. maximal electroshock Chemoconvulsants, e.g. pilocarpine, kainate, penthylenetetrazol, bicuculline, picrotoxin, flurothyl Genetic models, e.g. genetically epilepsy-prone rats, Mongolian gerbil, DBA/2J mice, photosensitive baboons, knockout mice
Absence seizure	Chemoconvulsants, e.g. low dose penthylenetetrazol, gamma-hydroxybutyrate Genetic models, e.g. genetic absence rats from Strasburg, Wistar Albino Glaxo/Rijswijk, tottering mice, stargazer mice, lethargic mice, slow-wave epilepsy mice, mocha mice, ducky mice

*Common animals used are rats and mice

Post-SE model with spontaneous recurrent seizure also comprises electrically induced models (e.g. electrical stimulation of hippocampus via perforant path, angular bundle or CA3 of ventral hippocampus and lateral or basolateral amygdala) and chemically induced models (e.g. pilocarpine or kainate) (13, 14, 146, 147). The spontaneous recurrent, partial and secondarily generalized seizures, damage of hippocampal and extrahippocampal, and alterations of behavior and cognition produced by the pilocar-pine- and kainate-induced models resemble clinical characteristics of TLE, hence, both are considered as representative models for TLE (32, 147).

**Challenges in animal models**

Undoubtedly, animal models have provided useful information in addressing critical research issues, including mechanism of epileptogenesis, which are impossible to study in humans due to ethical concerns. However, what is the degree that these models reflect the actual condition in humans with epilepsy? The actual mechanisms underlying epilepsy may be more complicated. For example, in TLE studies, post-SE models involved an acute triggering SE process that was frequently followed by a latency period with subsequent development of spontaneous motor seizure (148, 149) and hippocampal lesions similar to that showed in TLE patients (150-152). Nevertheless, these models cause neuronal loss not only in hippocampus and amygdale of the limbic area, but also extralimbic regions such as thalamus, hypothalamus and certain areas of celebral cortex, which are usually not involved in human TLE (147, 151). Hence, there is a need to develop new animal models that are able to reproduce unique characteristics of epilepsy as faithfully as possible and therefore, enhance the extrapolation of animal data to human condition. 

The animal models have a different etiologic process compared to status epilepticus in human, which is usually associated with traumatic brain injury and ischemic stroke (6, 9). Hence, it is difficult to justify whether animal models are able to ideally or adequately model human epileptogenesis process. Generally, it is believed that common mechanisms might underlie human and animal epileptogenesis (152). Another challenge for the animal model is related to various subtypes of either generalized or partial seizures in human epilepsy such as generalized myoclonic seizure, generalized atonic seizure and absence seizure (10). However, animal models usually manifest generalized tonic-clonic and limbic seizures only (140), which represent sub-population of patients with these types of epilepsy. Hence, this can limit current understanding on epileptogenesis in other epilepsy phenotypes. 

Application of the animal models in understanding other epileptic related conditions such as cognitive and behavioral changes could be improved. Many patients with TLE suffer from cognitive and behavioral alterations, such as depression, anxiety, psychosis and memory loss, which may be associated with morphologic and functional alterations in the temporal lobe (153). These behavioral alterations are difficult to evaluate directly in the current available models. Different tests have been designed to complement the assessment of behavioral alteration in animals, for instance, test for anxiety-related behavior (e.g. light/dark box test, elevated plus-maze and open-field tests), Irwin test for evaluating physiologic reflexes and behavioral abnormalities, and test for depression-like behavior (e.g. forced swimming test and tail suspension test) (154). These tests are useful for predicting the anxiety level in mice and provide insight into association between epilepsy and behavioral alterations.

There is a substantial degree of variability among individual animals in response to epileptogenic or convulsive stimuli in a specific model. In rodents for example, strain, gender and age factors affected the response to epileptogenic stimuli (151, 155, 156). Immature rats were more susceptible to status epilepticus than were adult rats, but immature hippocampi exhibit markedly less hippocampal damage and changes compared to adults after treatment with kainate or repeated kindling (157). Apart from age, different strains and genders may have different responses to epileptogenic stimuli. Female Sprague-Dawley rats were more sensitive to basolateral amygdala stimulation compared to Wistar rats in terms of status epilepticus induction and development of epilepsy after SE (156). Besides diversity in susceptibility to stimuli, strain factor also influences the patterns of hippocampal damage and neurodegeneration in affected brain areas (158). Such variability in animals may give results that cannot be reproduced from one laboratory to another and consequently, increases the difficulties in generalizing the findings in a specific model (159).

Currently, these animal models still remain valuable research tools in view of presence of an intact central nervous system, which provides an opportunity for studying the mechanism underlying epileptogenesis. There is no general agreement about which animal model may be most appropriate and relevant to human condition and none of the available models have been clinically validated (7). For a model to be validated, it should be highly predictive of clinical response, which is usually complicated by many forms of epilepsy with different pathophysiologies (141). Hence, to improve current animal models, the limitations and confounding factors should be taken into consideration in developing a novel animal model. 

## Conclusion

A greater understanding of pathogenesis of epilepsy will likely provide the basis fundamental for development of new antiepileptic therapies that aim to prevent the epileptogenesis process, or modify the progression of epilepsy in addition to treatment of epilepsy symptomatically. Animal models play a crucial and significant role in providing additional insight into mechanism of epileptogenesis, predo-minantly kindling and post-SE with spontaneous recurrent seizure models (7,140). With the help of these models, epileptogenesis process has been demonstrated to be involved in various molecular and biological pathways or processes. These include neurotransmission signaling pathway, molecular and genetic mechanism, neurogenesis and rewiring pathway, immunology and inflammatory pathway, apoptotic pathway, gene and protein regulation and other mechanisms that are not discussed in this article. However, it still remains unclear which mechanisms are required or necessary for genesis of epilepsy in human. Besides, there are some challenges that arise from the use of animal models in experimental studies, such as degree of representation from animal models to epileptic human models, different etiologies of seizure induction between animal models and human models, difficulty of evaluating behavioral alteration in animal models and variability among individual animals. In order to overcome these challenges, currently available animal models should be used with caution, validated and all the limitations and confounding factors should be taken into consideration. This could provide a more accurate picture of the epileptogenesis process and ultimately, contribute to development of new antiepileptogenic or disease-modifying therapies, which provide new hope for epileptic patients.
